# Crossing Treeline: Bacterioplankton Communities of Alpine and Subalpine Rocky Mountain Lakes

**DOI:** 10.3389/fmicb.2021.533121

**Published:** 2022-01-03

**Authors:** Kim Vincent, Hannah Holland-Moritz, Adam J. Solon, Eli M. S. Gendron, Steven K. Schmidt

**Affiliations:** ^1^Department of Ecology and Evolutionary Biology, University of Colorado, Boulder, CO, United States; ^2^Department of Molecular, Cellular, and Developmental Biology, University of Colorado, Boulder, CO, United States

**Keywords:** watershed connectivity, alpine, treeline, Rocky Mountain lakes, microbial communities, bacterioplankton, terrestrial-aquatic connections

## Abstract

From the aboveground vegetation to the belowground microbes, terrestrial communities differ between the highly divergent alpine (above treeline) and subalpine (below treeline) ecosystems. Yet, much less is known about the partitioning of microbial communities between alpine and subalpine lakes. Our goal was to determine whether the composition of bacterioplankton communities of high-elevation mountain lakes differed across treeline, identify key players in driving the community composition, and identify potential environmental factors that may be driving differences. To do so, we compared bacterial community composition (using 16S rDNA sequencing) of alpine and subalpine lakes in the Southern Rocky Mountain ecoregion at two time points: once in the early summer and once in the late summer. In the early summer (July), shortly after peak runoff, bacterial communities of alpine lakes were distinct from subalpine lakes. Interestingly, by the end of the summer (approximately 5 weeks after the first visit in August), bacterial communities of alpine and subalpine lakes were no longer distinct. Several bacterial amplicon sequence variants (ASVs) were also identified as key players by significantly contributing to the community dissimilarity. The community divergence across treeline found in the early summer was correlated with several environmental factors, including dissolved organic carbon (DOC), pH, chlorophyll-a (chl-a), and total dissolved nitrogen (TDN). In this paper, we offer several potential scenarios driven by both biotic and abiotic factors that could lead to the observed patterns. While the mechanisms for these patterns are yet to be determined, the community dissimilarity in the early summer correlates with the timing of increased hydrologic connections with the terrestrial environment. Springtime snowmelt brings the flushing of mountain watersheds that connects terrestrial and aquatic ecosystems. This connectivity declines precipitously throughout the summer after snowmelt is complete. Regional climate change is predicted to bring alterations to precipitation and snowpack, which can modify the flushing of solutes, nutrients, and terrestrial microbes into lakes. Future preservation of the unique alpine lake ecosystem is dependent on a better understanding of ecosystem partitioning across treeline and careful consideration of terrestrial-aquatic connections in mountain watersheds.

## Introduction

Mountain treelines mark the upper elevational limit of forest growth and an abrupt shift in ecosystem characteristics between forest vegetation and treeless tundra (Körner and Paulsen, 2004; Mayor et al., 2017). Forest growth at treeline can be limited by a variety of factors, such as heavy winds and unsuitable soils, but is most often temperature-limited (Smith et al., 2009). The precipitous transition from alpine (above treeline) to subalpine (below treeline) vegetation over a relatively short distance is clear. Less apparent is the partitioning of microbial communities in relation to treeline; yet, even soil bacteria and fungi of alpine and subalpine environments are distinct (Kernaghan and Harper, 2001; Thébault et al., 2014). Meanwhile, it is unclear to what extent microbial communities of alpine and subalpine lakes reflect the differences in community composition across treeline that their terrestrial counterparts do.

Mountain watersheds are like drains; and lakes are the recipients of the hydrologic inflow not only from upstream lakes, but also from the surrounding terrestrial environment (Baron and Campbell, 1997; Polis et al., 1997; Campbell et al., 2010). During spring snowmelt, water from melting snow moves downward across and through the landscape, interacting with the soil and flushing solutes and nutrients into lakes (Denning et al., 1991; Creed et al., 1996; Tsai et al., 2008; Fellman et al., 2009; Wilkinson et al., 2013). Aquatic bacterial communities are shaped by their chemical environment and could thus be influenced by the chemical content of the inflow (Crump et al., 2003; Fierer et al., 2007; Adams et al., 2010; Niño-García et al., 2016). Additionally, bacteria and other microbes inhabiting the snow, soil, and surfaces of plants and rocks are swept into lakes from the terrestrial environment (Mašín et al., 2003; Lindström et al., 2006; Crump et al., 2007; Ruiz-González et al., 2015, 2017; Hayden and Beman, 2016). Given this hydrologic connectivity with the surrounding divergent terrestrial environment across treeline, we hypothesized that bacterial communities of alpine and subalpine lakes would also be divergent.

Alpine lakes represent a unique and imperiled ecosystem whose sensitivity to environmental changes makes them a valuable tool for the early detection of changes in regional and global climates (Adrian et al., 2009; Williamson et al., 2009). Much of the responsiveness of these lakes can be attributed to the microbial communities dwelling within, who play an important role in the processing of nutrients and energy through the lake food web (Tranvik, 1988; Woodward et al., 2010). Shifts in bacterial community composition in response to their environment, such as changes to pH, temperature, and nutrient loading for example, are well documented (Adams et al., 2010; Sumampouw and Risjani, 2014; Niño-García et al., 2016; Glasl et al., 2019). Changes in community composition could have a cascade of consequences to higher trophic levels and alter the overall biogeochemical processing of the lake. Yet, research on high-elevation lakes in the U.S. Rocky Mountains can be challenging, often requiring multi-day field campaigns and traveling by foot over long distances while carrying heavy research and camping gear. The remoteness and challenging access terrain has left high-elevation lakes in this region relatively understudied.

Climate change induced alterations of high-elevation environments may lead to changes in the microbial community composition of mountain lakes. Changes to precipitation and snowpack (Williams et al., 1996; Trenberth, 2011; Gergel et al., 2017; Dong et al., 2019), can lead to an increase in springtime flushing of soil microbes, organic carbon, and nitrates (Sobek et al., 2005; Baron et al., 2009; Dong et al., 2019). And as the melting of glaciers advances (Paul et al., 2004; Moore et al., 2009), in increase in the influx of unique microbial communities from glacial meltwater is also likely. Additionally, treelines are encroaching upward worldwide (Lenoir et al., 2008; Harsch et al., 2009; Iverson and McKenzie, 2013). Over time, the increase in plant biomass in the terrestrial environment could lead to an increase in plant-derived nutrients washing into the lake. The combined effects of increased nutrient flushing, a greater influx of terrestrial microbes from soil and glacier melt, and the upward expansion of treeline, highlight the need to better understand how microbial communities of mountain lakes relate to treeline position to provide insights into how communities may change with altered terrigenous inputs in a changing climate.

Elevational patterns of life have long fascinated ecologists, but very few studies have compared microbial communities of aquatic alpine and subalpine lakes or examined elevational patterns of microbial community composition across treeline. Yet, interesting patterns have emerged from the few existing studies. For example, Teittinen et al. (2017) examined biofilms of ponds in Scandinavian mountains that spanned a low-elevation (10–1,038 m) Arctic treeline and found differing patterns of richness for autotrophic and heterotrophic microbes. Specifically, bacterial (excluding cyanobacteria) richness decreased as elevation increased, but the richness of diatom and cyanobacteria (autotrophic) was higher at mid-elevations than both high and low elevations (i.e., demonstrating unimodal elevational patterns). In the California Sierra Nevada, Nelson (2009) examined bacterial community composition between inlet streams and lakes, discovering headwater inlet streams to be consistently distinct from all downstream samples regardless of the location of treeline. To our knowledge, only one study has made direct comparisons of bacterioplankton communities of alpine and subalpine lakes. Bastidas Navarro et al. (2014) found bacterioplankton communities in North Patagonia Andean lakes (1,380–1,950 m) to be distinct across a deciduous treeline in mid-austral summer. Thus, despite the obvious distinctions between alpine and subalpine terrestrial environments, very little is known about the partitioning of aquatic communities across treeline. This paper represents the first study to make the comparison of bacterioplankton communities across treeline in a high-elevation (ranging from 3,021 to 3,529 m) coniferous environment.

The main goal of this study was to determine whether bacterioplankton community composition (identified with 16S rDNA SSU amplicon sequencing) of alpine lakes differed from subalpine lakes in a high-elevation coniferous landscape. We hypothesized that bacterial community composition would be distinct in both the early and late summer based on previous research demonstrating (1) lake chemistry differs across treeline (Duff et al., 1998; Clow and Sueker, 2000); (2) aquatic bacterial communities are shaped by their chemical environment (Crump et al., 2003; Fierer et al., 2007; Adams et al., 2010; Niño-García et al., 2016); (3) terrestrial microbes that differ across treeline (Kernaghan and Harper, 2001; Thébault et al., 2014) could wash into the lakes; and (4) bacterioplankton communities of Patagonian lakes differ across treeline in mid-austral summer (Bastidas Navarro et al., 2014). To our knowledge, the microbial communities of the lakes in the present study have not yet been researched. Thus, in addition to determining landscape-level partitioning of communities across treeline at two time points (early and late summer) and the abiotic factors that may be driving community differences, this study provides valuable identification of the bacterial communities that inhabit high-elevation lakes in this region.

## Materials and Methods

### Study Lake Descriptions

**Figure 1:**
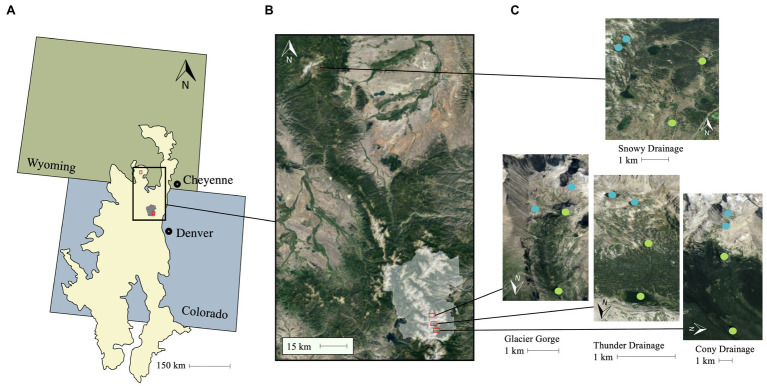
Study lake locations. All lakes are located in the Southern Rocky Mountain Ecoregion (US Environmental Protection Agency, 2013), represented by the cream polygon spanning Wyoming and Colorado in **(A)**. **(B)** Shows an inset of the area encapsulating the watersheds, with the Rocky Mountain National Park boundary in white and each of the watersheds outlined with a red box. **(C)** Shows alpine lakes marked with blue circles and subalpine lakes marked with green circles overlayed on satellite imagery (sourced from Google Maps) for each watershed. The northern most survey watershed is located in the Snowy Range Mountains west of Cheyenne, WY.

We sampled a total of 16 lakes in the Southern Rocky Mountain ecoregion (US Environmental Protection Agency, 2013), ranging in elevation from 3,021 to 3,529 m (Figure 1; Table 1). The maximum depth of the lakes varied from 1.8 to 36 m (Table 1), but depth did not differ between alpine and subalpine lakes (*p* > 0.05), and both shallow and deep lakes were represented across alpine and subalpine treatments (Table 1). Each of the four watersheds had two alpine and two subalpine lakes, which are hydrologically connected through the main inlet and outlet streams, ephemeral streams, surface runoff, and/or groundwater. Three of the watersheds were located in Rocky Mountain National Park (RMNP), Colorado; and the fourth was located in the Snowy Range of southern Wyoming. The Snowy Range watershed was chosen for comparison with RMNP lakes to determine whether the pattern held in different geographic locations. We designated lakes as alpine or subalpine with aerial photos prior to sampling. The treelines of some drainage are more abrupt than others, but position in relation to treeline was easily verified in the field. Shrubs and tundra vegetation are present in the terrestrial environment surrounding the alpine lakes in all four watersheds. Forest vegetation around treeline is dominated by Engelmann spruce (*Picea engelmannii*) and lodgepole pine (*Pinus contorta*).

**Table 1:** General characteristics of study lakes.

Lake Name	Elevation (m)	Max. Depth (m)	Temp. (C)	Fish Presence	Geographic Coordinates: Lat, Long	Date Visit 1	Date Visit 2	Date of Ice-off
**Alpine Lakes**									
Blue Lake	3,408	9.1	7.5	Fishless	40.267991, −105.631724	7/23/16	8/25/16	NA
Upper Hutchinson	3,412	3.6	9.0	Fishless	40.173843, −105.647759	7/18/16	8/22/16	NA
East Glacier	3,412	7.2	11.6	Fish	41.377876, −106.255630	7/26/16	8/28/16	NA
Lion Lake 2	3,469	11.4	8.4	Fishless	40.237771, −105.641617	7/11/16	8/15/16	NA
West Glacier Lake	3,470	6.2	12.0	Fish	41.377270, −106.259020	7/26/16	8/28/16	between 7/19/16 and 7/25/16
Cony Lake	3,508	19.2	7.8	Fishless	40.172982, −105.657991	7/18/16	8/22/16	between 7/15/16 and 7/17/16
Snowbank Lake	3,512	8.5	8.2	Fishless	40.240285, −105.644596	7/11/16	8/15/16	between 7/6/16 and 7/10/16
Frozen Lake	3,529	27.0	4.2	Fishless	40.257713, −105.642722	7/23/16	8/25/16	7/23/16
**Subalpine Lakes**									
Finch Lake	3,021	3.2	17.9	Fish	40.183410, −105.593179	7/19/16	8/23/16	NA
Mills Lake	3,030	8.4	15.9	Fish	40.289596, −105.641664	7/23/16	8/25/16	NA
Jeep Lake	3,108	1.7	19.4	Fishless	41.355507, −106.272790	7/27/16	8/29/16	NA
Thunder Lake	3,225	16.2	9.2	Fish	40.222204, −105.647169	7/12/16	8/16/16	NA
Pear Reservoir	3,225	11.3	14.0	Fish	40.176333, −105.626098	7/19/16	8/23/16	NA
Little Brooklyn	3,225	2.1	19.3	Fish	41.361910, −106.245970	7/27/16	8/29/16	NA
Black Lake	3,236	36.0	9.2	Fish	40.265354, −105.641260	7/23/16	8/25/16	NA
Lion Lake 1	3,373	1.8	11.5	Fishless	40.231947, −105.638670	7/12/16	8/16/16	NA

The lake type indicates whether the lake is located above or below treeline, and the Park/Range variable refers to whether the lake is located in Rocky Mountain National Park (RMNP) or the Snowy Range. Max depth refers to the maximum depth recorded in the field and averaged between both early summer (Visit 1) and late summer (Visit 2). The surface temperature was taken at the time of sampling and also averaged between both visits.

### Sample Collection

During the summer of 2016, we sampled each lake twice during the ice-free season (approximately June to September) for a total of 32 lake visits (specific dates listed in Table 1). The first of the two sampling trips was made in July as early as possible after ice-off (the date when the lake is 100% free of ice) to safely access the uppermost lake in each watershed. The second sampling trip was made approximately 5 weeks after the first visit (in August). The majority of these lakes (14/16) are located several kilometers into the backcountry (no vehicle access) and require overnight visits with camping permits secured months in advance. Because the lakes melt before the surrounding catchment is snow-free, travel through snow was often required for the first visit. Substantial springtime avalanche danger, catchment snow cover, and the remoteness of these lakes limited reconnaissance trips to identify the precise date of ice-off for every lake. However, through the limited reconnaissance trips and communication with other limnologists in the area, we were able to confidently determine the date of ice-off for the uppermost lake in each watershed within 1 week (listed in Table 1). Ice meltout (the thawing period for a lake) can be quite rapid at the time of ice-off. In fact, we witnessed the precise moment when the last piece of ice melted from Frozen Lake, the uppermost lake in the Glacier Gorge drainage, on July 23, 2016. All four of the lakes within a watershed were sampled in as short of a window as possible to minimize temporal effects (within 36 h from one another). We additionally restricted sampling to the hours of 08:00 and 13:00 to limit diurnal changes and safely avoid afternoon thunderstorms.

Prior to leaving for the field, we sterilized 500-ml high-density polyethylene (HDPE) sample collection bottles and the field filtering apparatus by triple rinsing with 10% HCl solution and nano-pure DI water. The sample bottles were triple-rinsed with sample water before the final sample was collected. Glass sample bottles were avoided in the field because of the potential to break in overnight packs. The clear acrylic Van Dorn was soaked for 24 h in nano-pure DI water and tripled-rinsed in nano-pure DI water (HCl solution was not used to avoid damage to the Van Dorn). We packed in research and camping gear by foot on day 1 (up to 11 km from the trailhead) to designated permitted backcountry camping spots in the National Park that we used as base camp. At sunrise the following day, we hiked from base camp to the uppermost lake in the watershed (generally 3–5 km off-trail hiking from base camp). We paddled to the deepest point of the lake with an inflatable Alpacka® raft and recorded surface water temperature and lake depth with a Signstek® FF-03 depth finder. To maintain a safe pack weight and keep the cost of analyses down, we limited our sample collection to one per lake. Yet, because some of the deeper lakes are likely to stratify by the end of the summer (Lewis, 1983) and microbial community composition often diverges with stratification (Shade et al., 2008), we opted to mix surface and bottom samples to better represent the communities of the lake. Thus, we collected a sample from the surface of the lake as a grab sample and another from approximately 1 m above the bottom of the lake with the Van Dorn and later mixed them during filtering. Samples were collected using the same protocol at all lakes regardless of depth and the potential for stratification. All field sampling was performed under scientific permit ROMO-2016-SCI-0014.

### Sample Processing

After returning to base camp, we triple-rinsed the filtering apparatus with DI water, rinsed once with the sample water, and then poured even volumes of the surface and the bottom sample into the filtering apparatus. We hand-filtered between 500 and 1,000 ml lake water through 0.7-μm pore size glass fiber filters (GF/F, pre-combusted at 475°C for 4 h) and 500 ml though 0.2-μm pore size sterile filters. The filtrate (0.7-μm pore size filter) was transferred to pre-sterilized 500 ml HDPE bottles and kept for water chemistry characterization. We used forceps to transfer the 0.7-μm GF/F filters [chlorophyll-a (chl-a) samples] and 0.2-μm filters (microbial samples) to sterile plastic vials. Forceps and the filtering apparatus were rinsed three times with DI water and sample water between samples. To minimize photosynthesis and microbial activity, samples were stored in a black plastic bag submerged in a cold shaded creek until transfer to the laboratory was possible (within a maximum of 36 h after collection).

### Chemical Analyses

We characterized the chemical properties of the water samples at the University of Colorado (Boulder, CO). Immediately upon arrival to the laboratory, we allowed a subset of each sample to reach room temperature, recorded pH with an Oakton benchtop pH meter, and analyzed ammonium (NH_4_^+^) using a discrete chemical analyzer (SmartChem 170). The remaining filtrate was frozen (−20°C) for a maximum of 3 months until analysis for total dissolved nitrogen (TDN), total dissolved phosphorus (TDP), and total dissolved organic carbon (DOC). We analyzed TDP colorimetrically (Grasshoff et al., 2009), TDN with an ion chromatograph, and DOC with a Shimadzu 7100 carbon analyzer. We extracted chl-a from the 0.7-μm GF/F filters using the 90% hot ethanol method (Nusch, 1980), then covered samples in foil, and kept refrigerated (4°C) until analysis 24 h later. We then hand-filtered extracted samples through Whatman GF/C (pore size 1.2 μm) filters before spectrophotometric analysis and corrected final values for phaeopigment.

### Microbial Community Analyses

Microbial communities were identified using high-throughput amplicon sequencing. We extracted genomic DNA from the samples according to the manufacturer’s protocol for the PowerWater® DNA Isolation Kit (MoBio Inc., Carlsbad, CA, United States) and used PCR to amplify the V4-V5 region of bacterial 16S rRNA genes using the 515 forward (GTGYCAGCMGCCGCGGTAA) and 806 reverse (GGACTACNVGGGTWTCTAAT) primer set (Caporaso et al., 2012). To quantify and control for bacterial contamination during processing, we included a no-template control of DNA-free ultrapure water in our extraction and amplification steps. Sequencing of all 32 samples and the no-template control was completed at the BioFrontiers Sequencing Core Facility at the University of Colorado at Boulder using Illumina MiSeq paired-end technology (2 × 250 bp with V2 chemistry).

### Bioinformatics

All bioinformatics and statistical analyses were completed using R version 4.0.3 (R Development Core Team, 2019). Demultiplexing, quality filtering, denoising, merging of paired-end reads, singleton and chimera removal, and taxonomic assignment of the raw 16S rRNA gene sequences were completed using the DADA2 pipeline v 1.16 (Callahan et al., 2016). Sequences were identified as unique amplicon sequence variants (ASV) based on single nucleotide differences. ASVs are quickly gaining popularity for defining unique sequences, and numerous recent studies have been published using this technique in lake communities (e.g., Kraemer et al., 2020; Mayr et al., 2020). Similar ecological results are obtained with both traditional OTUs and ASVs (Glassman and Martiny, 2018), but ASVs have advantages in reusability, reproducibility, and comprehensiveness in sequence identification (Callahan et al., 2017).

Defaults of the DADA2 pipeline were used throughout, unless explicitly specified. Primers were first removed with Cutadapt v. 3.1 (Martin, 2011), but because the quality of the reads dropped slightly on the left ends of the reads, we trimmed the left end of the forward reads at 19 bps and reverse reads at 20 bps to ensure that any potential residual primers were removed. We set a minimum length of 50 bps and trimmed the right end of forward reads at 100 bps and reverse reads at 110 bps. The max error rate was set at 1, and the minimum quality score was set to 11. We assigned taxonomy by aligning remaining sequences with the SILVA version 138 database (Quast et al., 2012; Callahan, 2020). After quality filtering, merging of forward and reverse reads, and removing chimeras through the DADA2 pipeline, 75.43% of reads remained. We then removed chloroplasts, mitochondria, eukaryotes, two genera of common human contamination, *Haemophilus* spp. and *Neisseria* spp. (Sheik et al., 2018), and taxa without assignment at the domain level with the function filter_taxa_from_input() (R package *mctoolsr*, https://github.com/leffj/mctoolsr/). The abundance of remaining sequences was standardized through rarefaction before further analysis to a depth of 8,999 reads, the minimum number of reads in the dataset. The blank (5,813 reads) was dropped from the dataset at rarefaction. We also verified taxonomic assignment for the top 10 most abundant bacteria and any ASVs identified in community analyses as important drivers of community composition by comparing against the NCBI-BLAST database (Altschul et al., 1990). All taxa are reported throughout the manuscript to the lowest resolution after NCBI-BLAST database verification. Raw sequence reads were deposited to NCBI (accession #PRJNA604620).

### Data Analyses

We used the R package *mctoolsr*^1^ for basic descriptions of the bacterial dataset and *tidyverse* (Wickham and Chang, 2013) for the majority of data processing and filtering. Microbial community analyses were conducted with the R package *vegan* (Oksanen et al., 2018). Before comparing the water chemistry variables by lake type and visit using multivariate ANOVA (MANOVA), we tested the distribution for each variable with the Shapiro-Wilk’s test and log-transformed variables with a non-normal distribution. Chemistry variables were then tested for collinearity using Pearson’s correlation, and variables with the highest coefficient of determination (*R*^2^) out of the correlating variables were kept for future analyses. We also used Akaike’s information criterion (AIC) to compare the relative importance of regression models created for the collinear variables. We tested the differences in abundance between lake types and early and late summer visits for the top 10 most abundant taxa and the ASVs significantly contributing to the community dissimilarity using the Kruskal-Wallis rank sum test. To assess differences in microbial community composition between alpine and subalpine lakes, we calculated Bray-Curtis dissimilarities and tested differences using permutational MANOVA (PERMANOVA). Early summer and late summer visits were analyzed separately, and because bacterial communities differed by watershed, we included watershed as a blocking variable. We tested for dispersion differences using the function betadisper() (R package, *vegan*). We also performed a follow-up similarity percentage analysis (SIMPER) with the function simper() (R package, *vegan*), to identify the key species contributing to the community dissimilarity (statistically significant community composition differences between groups). SIMPER analysis performs pairwise comparisons to identify species contributing at least to 70% of the differences between groups of a Bray-Curtis dissimilarity matrix and indicates in which group it is more abundant. We additionally tested for correlations between water chemistry properties and community dissimilarities on the early summer visit (when community dissimilarity was significant) by overlaying an non-metric multidimensional scaling (NMDS) ordination using the function envfit() (R package, *vegan*). To visualize differences among community composition of alpine and subalpine lakes, we constructed NMDS ordinations based on Bray-Curtis distance matrices using the function calc_ordination() (R package, *mctoolsr*). Figures were created with the R package, *ggplot2* (Wickham and Chang, 2013) and edited with Microsoft PowerPoint.

## Results

### General Description of Bacterial Dataset

**Figure 2:**
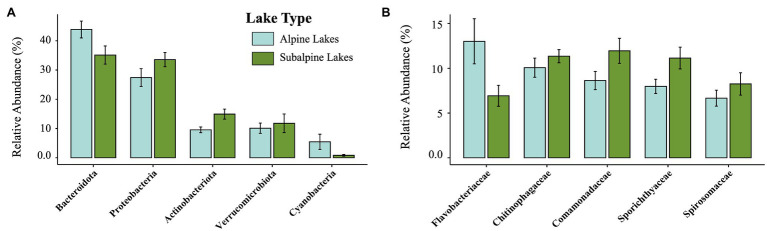
Relative abundance of the top five most abundant taxa by phylum **(A)**, and family levels **(B)**, comparing alpine (light blue) and subalpine (green) lakes.

**Figure 3:**
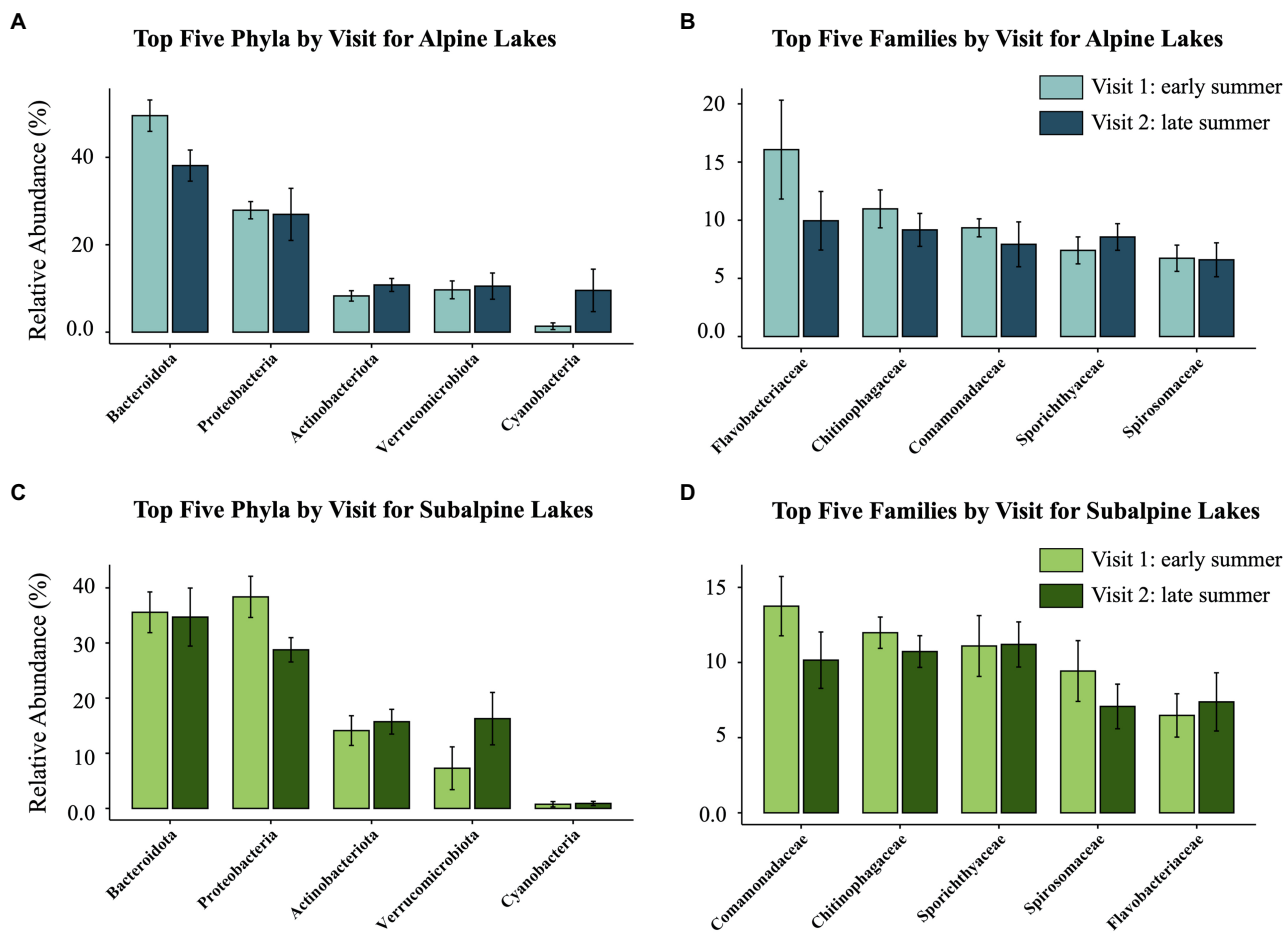
The relative abundance of the top five most abundant taxa compared between the early summer (Visit 1) and late summer (Visit 2) visits for both alpine (blue, above) and subalpine (green, below) lakes. The two figures on the left **(A,C)** represent the top five phyla, and the two figures on the right **(B,D)** represent the top five families for each lake type (alpine or subalpine).

After filtering the sequence dataset, 287,968 reads from 1,441 ASVs remained. The top 10 most abundant ASVs overall made up 25.02% of the total reads. The five most dominant phyla (Figures 2, 3) found across all samples include the following: Bacteroidota (39.6% of reads), Proteobacteria (30.5% of reads), Actinobacteria (12.2% of reads), Verrucomicrobia (10.9% of reads), and Cyanobacteria (3.1% of reads). The five most dominant bacterial families (Figures 2, 3) found across all samples include (in order of relative abundance): Chitinophagaceae (10.7% of reads), Comamonadaceae (10.4% of reads), Flavobacteriaceae (10.0% of reads), Spirochaetaceae (9.5% of reads), and Spiromaceae (7.5% reads).

### Microbial Comparisons Across Treeline

**Figure 4:**
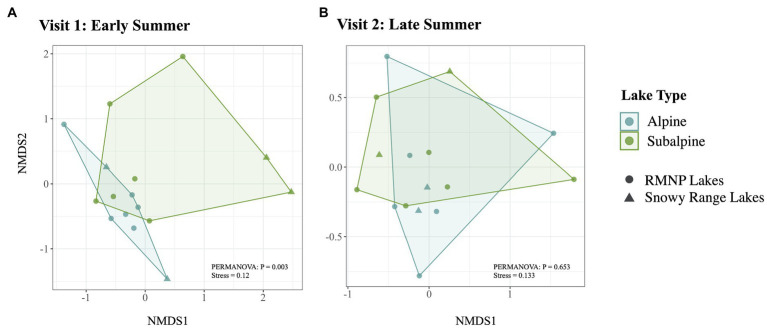
Bacterial community composition is represented for alpine (in blue) and subalpine (in green) lakes for early summer (Visit 1, **A**) and late summer (Visit 2, **B**) with non-metric multidimensional scaling (NMDS) Bray-Curtis ordination. Alpine lake bacterial community composition differs from that of subalpine lakes on Visit 1 in the early summer (*p* = 0.003), but does not differ on Visit 2, by the end of the summer (*p* = 0.653).

**Figure 5:**
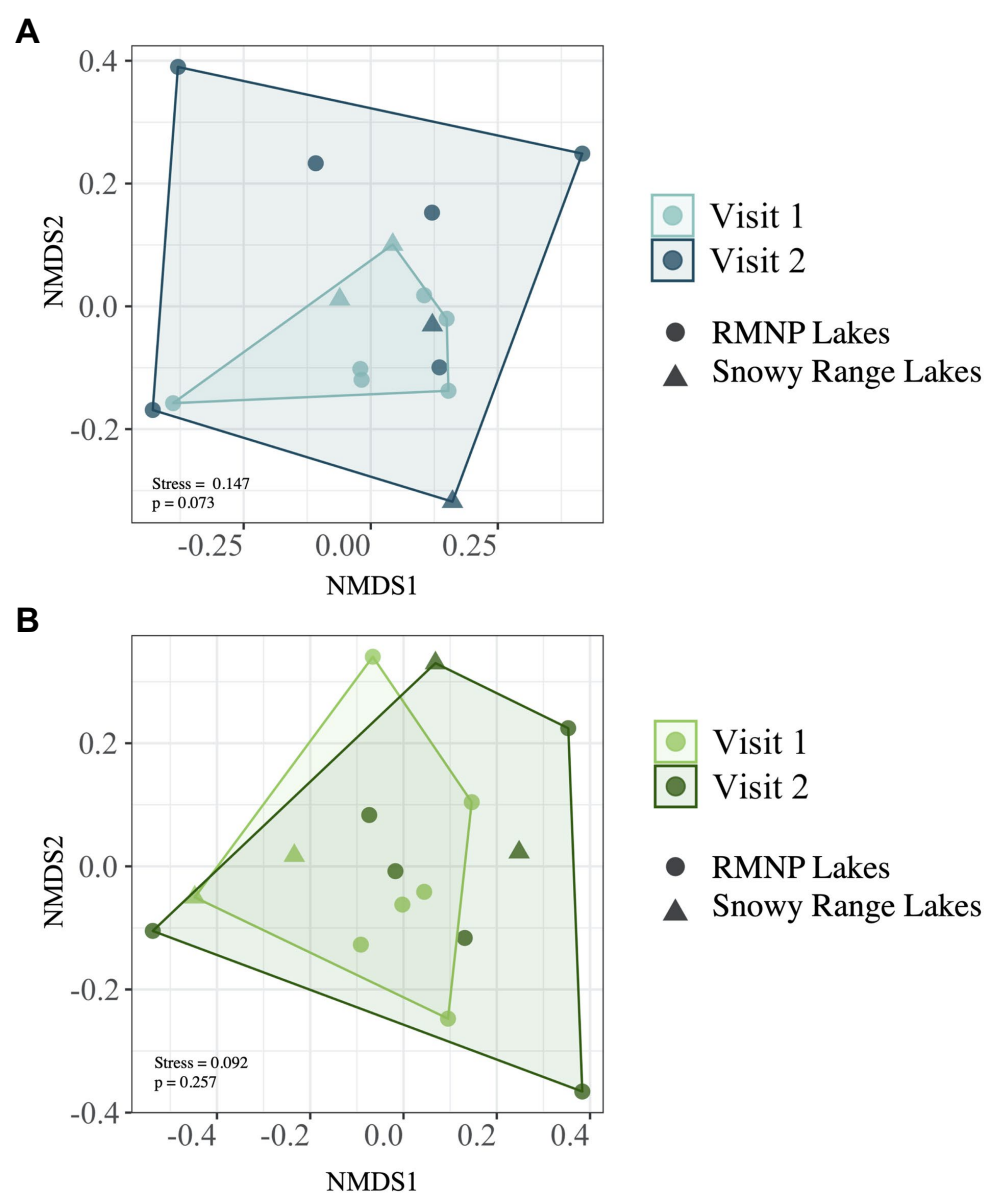
Dispersion of bacterial communities on the early summer (Visit 1) and late summer (Visit 2) visits separated for alpine **(A)** and subalpine lakes **(B)**. Alpine lakes are displayed in two shades of blue: light blue on Visit 1 and dark blue on Visit 2. Subalpine lakes are shown in two shades of green: light green on Visit 1 and dark green on Visit 2. While the dispersion is greater on Visit 2 for both alpine and subalpine lakes, the differences were not statistically significant (*p* = 0.073 and *p* = 0.257 for alpine and subalpine lakes, respectively).

Of the top 10 most abundant ASVs (Table 2) across all samples, two were more abundant in one of the two lake types. *Candidatus Planktophila sulfonica* (ASV_8) was found to be more abundant in alpine lakes (*p* = 0.008), and *Polynucleobacter asymbioticus* sp. (ASV_4) was found to be more abundant in subalpine lakes (*p* = 0.008). Bacterial communities of alpine lakes differed from subalpine lakes in the early summer when blocked by watershed (*p* = 0.003, *R*^2^ = 0.114, Figure 4A), and differences were not due to differences in dispersion (*p* = 0.774). This community dissimilarity (difference in community composition) is based on both richness or number of ASVs, and evenness, the degree to which the abundance of each ASV is similar. Comparisons between alpine and subalpine lake communities were blocked by watershed because the bacterial communities of at least one watershed differed from the others on both of the visits (early summer visit *p* = 0.018, *R*^2^ = 0.264; late summer visit *p* = 0.005, *R*^2^ = 0.274). By the second visit, bacterial communities of alpine and subalpine lakes were clearly not different from one another (*p* = 0.653, Figure 4B). Also notable, the dispersion of the community composition appears larger on the second visit for both alpine and subalpine lakes (Figure 5), but not significantly so for alpine (*p* = 0.073, Figure 5A) or subalpine lakes (*p* = 0.257, Figure 5B). Thus, bacterial community composition differed across treeline in the early summer, but not by late summer.

**Table 2:** The top 10 most abundant amplicon sequence variants (ASVs) in the dataset are reported to the lowest taxonomic resoluBon known. The abundance of Polynucleobacter asymbioBcus (ASV_4) was associated with subalpine lakes (*p* < 0.05) and Candidatus Planktophila sulfonica (ASV_8) was associated with alpine lakes (*p* < 0.05).

Top 10 most abundant ASVs	Mean number of reads		Lake type association
	Alpine	Subalpine	
*Pseudarcicella* sp. ASV_1	326.94	582.63	NA *p* = 0.105
*Flavobacterium* sp. ASV_2	498.50	140.56	NA *p* = 0.093
*Sediminibacterium* sp. ASV_3	220.31	247.63	NA *p* = 0.850
*Polynucleobacter asymbioticus* ASV_4	138.56	308.56	Subalpine^*^ p = 0.008
Sporichthyaceae ASV_5	168.31	251.50	NA *p* = 0.118
*Acidovorax* sp. ASV_6	135.81	287.50	NA *p* = 0.09
*Sediminibacterium* sp. ASV_7	177.63	178.69	NA *p* = 0.447
*Candidatus Planktophila sulfonica* ASV_8	94.56	208.50	Alpine^*^ p = 0.008
*Flavobacterium* sp. ASV_9	210.06	65.75	NA *p* = 0.936
env OPS 17 ASV_10	198.69	52.00	NA *p* = 0.651

The mean number of reads and lake type associations are included. ^*^Significant correlations (*p* < 0.05, Kruskal-Wallis rank sum test) of ASV abundance with lake type are displayed in red and starred.

### Key Species

A total of 11 ASVs were identified as playing a significant role in the community dissimilarity between alpine and subalpine lakes found on the first visit (Table 3); seven were more abundant in alpine lakes, and four were more abundant in subalpine lakes. Listed in order of their contribution to the community dissimilarity, the substantial players associated with alpine lakes include the following: (1) *Acinetobacter bereziniae* (ASV_288); (2) a member of the family Oxalobacteraceae (ASV_250); (3) a member of the genus *Flavobacterium* (ASV_122), (4) *Piscinibacter* (ASV_74), (5) *Caulobacter* (ASV_47), and (6) *Pedobacter* (ASV_82); and finally (7) a member of the family, NS11-12 marine group (ASV_20). The abundance of all seven of the alpine-associated ASVs was greater in alpine lakes than subalpine lakes (*p* < 0.05), and *Acinetobacter bereziniae* (ASV_288) was never detected in a subalpine lake. The four ASVs associated with subalpine lakes that were major players in the community dissimilarity between alpine and subalpine lakes included the following: (1) a member of the order Microtrichales (ASV_131), (2) a member of the family NS9 marine group (ASV_100), (3) *Polynucleobacter asymbioticus* (ASV_4), and (4) a member of the genus *Pseudarcicella* (ASV_1). The abundance of all but one of the subalpine-associated ASVs was significantly higher in subalpine lakes than alpine lakes (*p* = 0.104 for ASV_131, *p* < 0.05 for the remaining). The abundance of the Microtrichales member (ASV_131) was higher in subalpine lakes, but not significantly so (*p* > 0.05). NS9 marine group (ASV_100) was never detected in an alpine lake.

**Table 3:** Mean abundance by lake type and relationships between chemistry variables for ASVs identified as significantly (*p* < 0.05) contributing to at least 70% of the early summer community dissimilarity between alpine and subalpine lakes.

Lowest taxonomic resolution	Mean no. reads Visit 1		DOC		pH			chl-a		TDN	
	Alpine	Subalpine	Visit 1	Visit 2	Visit 1		Visit 2	Visit 1	Visit 2	Visit 1	Visit 2
Alpine-associated ASVs											
*Acinetobacter bereziniae* ASV_288	17.87	0	0.003^*^ r = −0.70	-	-		-	-	-	-	-
Oxalobacteraceae ASV_250	24.63	3.38	0.023^*^ r = −0.56	-	-		-	-	-	0.042^*^r = −0.48	-
*Flavobacterium* sp. ASV_122	49.88	4.38	0.058	-	-		-	-	-	0.073	-
*Piscinibacter* sp. ASV_74	67.35	21.75	0.027^*^ r = −0.55	-	-		-	-	0.004^*^ r = 0.67	-	-
*Caulobacter* sp. ASV_47	102.63	35.88	0.017^*^ r = −0.59	-	0.033^*^ r = −0.54		0.010^*^ r = −0.63	-	-	0.014^*^r = −0.60	-
*Pedobacter* sp. ASV_82	88.0	8.5	0.002^*^ r = −0.71	-	-		-	-	0.058	-	-
NS11-12 marine group ASV_20	151.0	31.38	0.024^*^ r = −0.56	0.021^*^ r = −0.57	-		-	-	-	-	-
Subalpine-associated ASVs											
^**^Microtrichales ASV_131	4.13	25.63	-	0.073	-	0.020^*^ r = −0.58	-	-	-	-	
NS9 marine group ASV_100	0	32.38	0.010^*^ r = 0.62	-	-	-	-	-	-	-	
*Polynucleobacter asymbioticus* ASV_4	149.25	308.38	-	-	-	-	-	-	-	-	
*Pseudarcicella* sp. ASV_1	295.88	752.25	-	-	-	-	< 0.001^*^ r = −0.75	-	-	-	

** ASV_131, the member of Microtrichales, was the only ASV to not also be correlated to lake type (*p* > 0.05, Kruskal-Wallis rank sum test).

Also included are the mean number of reads per lake type on the early summer visit (Visit 1) and the *p* values and correlation coefficients for select chemistry variables. The chemistry variables shown include those that were found to significantly contribute to community dissimilarity (through envfit analysis) and include dissolved organic carbon (DOC), pH, chlorophyll-a (chl-a), and total dissolved nitrogen (TDN). Values of *p* < 0.1 are noted and those with ^*^significant values at the *p* < 0.05 level are starred and printed in red.

### Abiotic Factors Correlated With Community Dissimilarity

**Figure 6:**
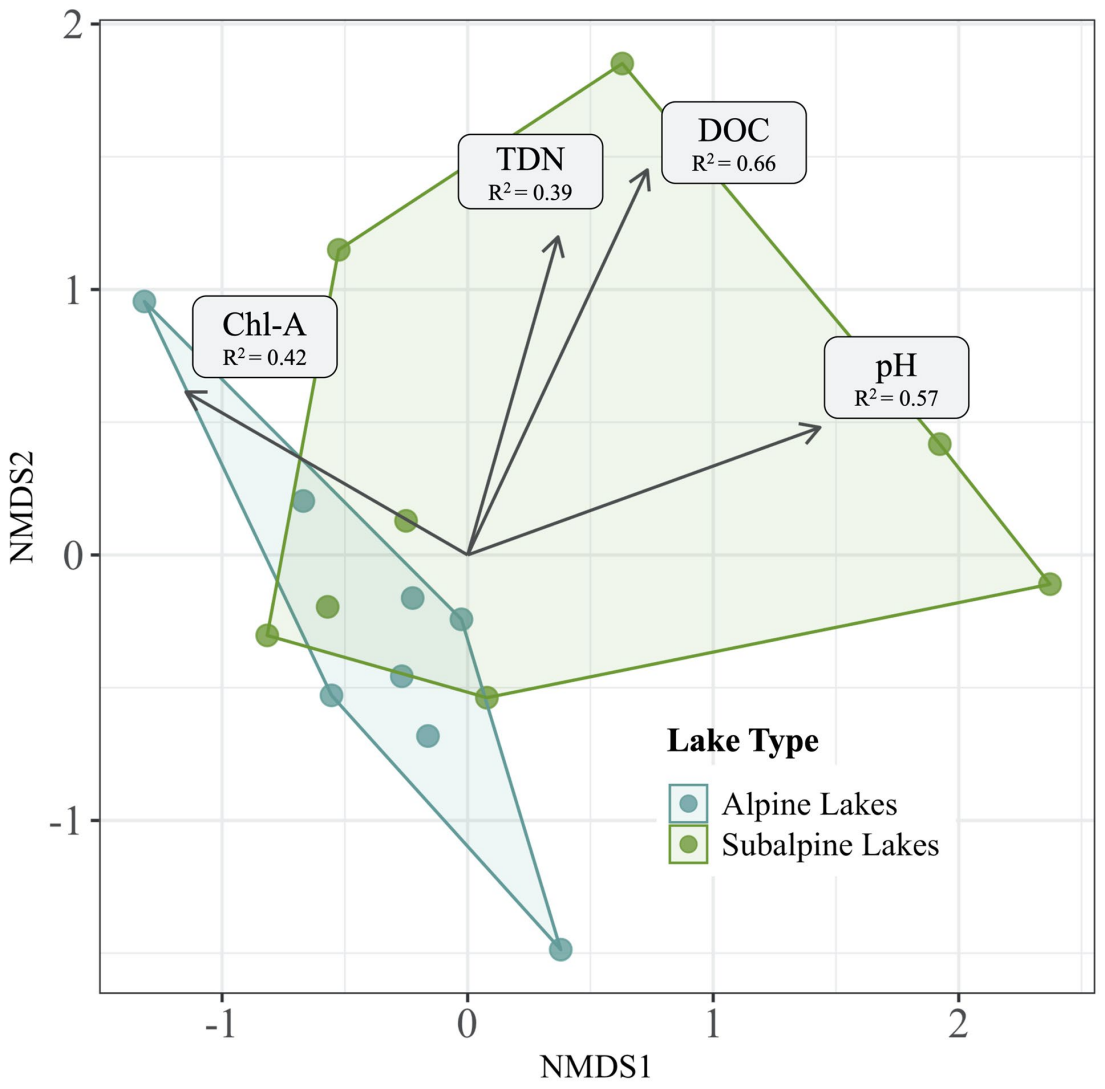
Abiotic factors significantly correlated (*p* < 0.05) with community dissimilarity of the early season are overlaid over an NMDS Bray-Curtis ordination plot displaying alpine lake communities in blue and subalpine lake communities in green. Factors significantly correlated with community dissimilarity include pH, DOC (dissolved organic carbon), chl-*a* (chlorophyll-*a*), and TDN (total dissolved nitrogen).

**Figure 7:**
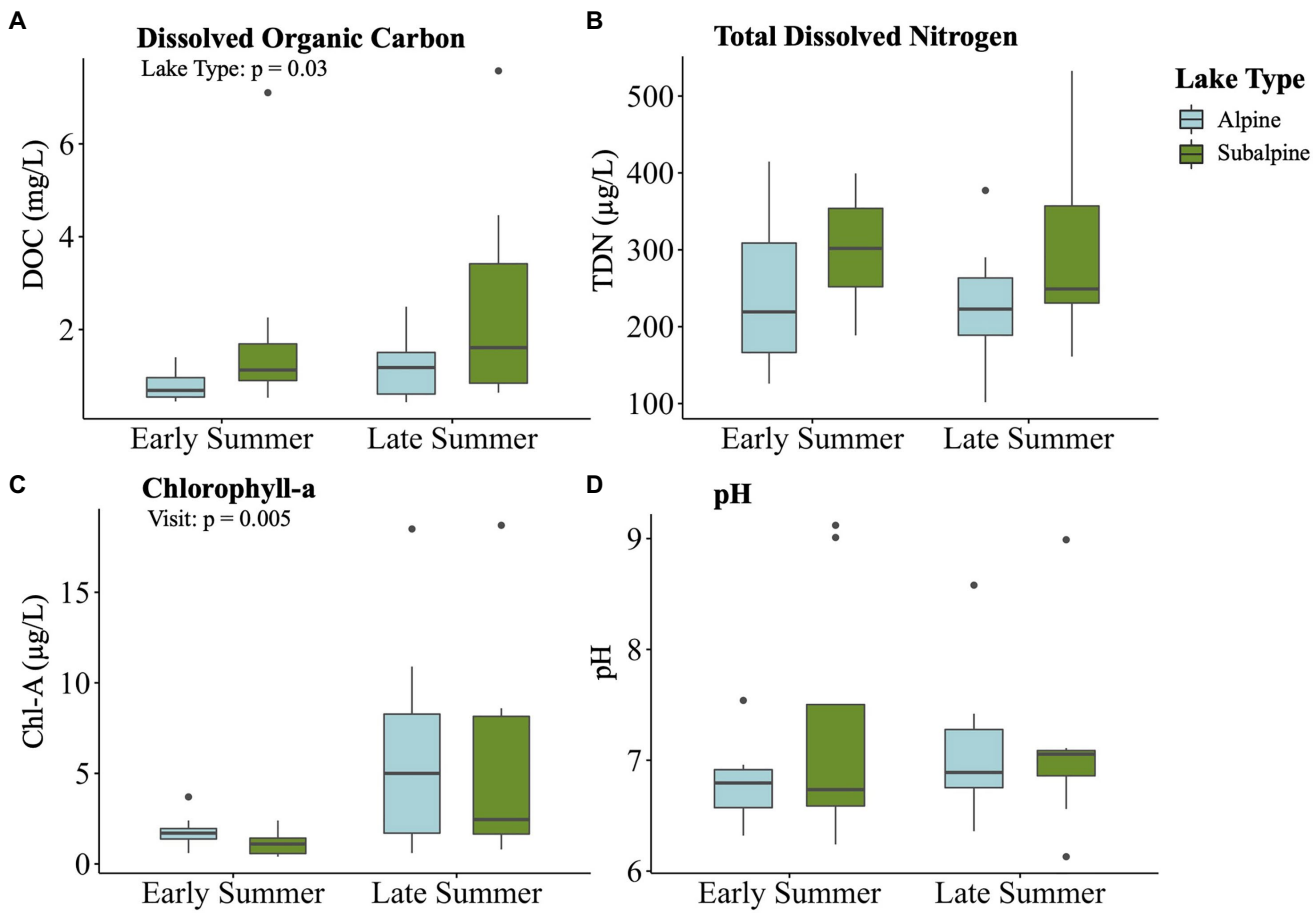
Boxplots displaying the four water chemistry variables that significantly correlated with community dissimilarity on the first visit by visit and lake type: **(A)** Lake surface temperature, **(B)** total DOC, **(C)** total dissolved nitrogen (TDN) and **(D)** pH. Alpine lakes are shown in blue, and subalpine lakes are shown in green.

After variables were reduced to avoid collinearity, all four of the remaining variables (DOC, pH, chl-a, and TDN) were significantly correlated with the bacterial community dissimilarity (*p* < 0.05 for all) across treeline (Figure 6). DOC had the highest correlation with community dissimilarity between alpine and subalpine lakes on in the early summer (*R*^2^ = 0.66), followed by pH (*R*^2^ = 0.57), chl-a (*R*^2^ = 0.42), and finally TDN (*R*^2^ = 0.39). Only two abiotic factors differed between alpine and subalpine lakes or early summer and late summer visits (Figure 7). The concentration of DOC was higher in subalpine than alpine lakes (*p* = 0.030, mean alpine = 1.03 mg/L ± 0.61 SD, mean subalpine = 2.24 mg/L ± 2.24 SD) and was the only abiotic factor that differed by lake type. Chlorophyll-a was the only abiotic factor that differed by visit; concentrations were higher in the late summer than the early summer (*p* = 0.005, mean early summer = 1.5 μg/L ± 0.89 SD, and mean late summer = 5.9 μg/L ± 5.92 SD).

### Key Species’ Response to Abiotic Factors

All of the major players listed above were significantly correlated with the community dissimilarity between alpine and subalpine lakes, but had varying responses to the chemistry variables. *Caulobacter* sp. (ASV_47) was also found in greater abundance in acidic lakes in both the early and late summer (*p* = 0.033 and *p* = 0.010, respectively). Both *Caulobacter* sp. (ASV_47) and a member of Oxalobacteraceae (ASV_250) were negatively correlated with TDN in the early summer and in the late summer. Not only did DOC have the highest correlation with community dissimilarity between alpine and subalpine lakes, it was also correlated with the most of the ASVs identified as major players in the community dissimilarity out of any of the contributing chemistry variables (Table 3). As expected, all of the alpine-associated ASVs that were significantly correlated with DOC were more abundant in lakes that were lower in DOC; while the subalpine-associated ASV, NS9 marine group (ASV_100) was more abundant in lakes with higher DOC concentrations. The alpine-associated ASV, NS11-12 marine group member (ASV_20) was the only ASV correlated with DOC on both the first and the second visits. It also contributed the least to the community dissimilarity out of all of the alpine-associated ASVs identified. *Pseudarcicella* sp. (ASV_1) was the most abundant ASV in the dataset and was positively correlated with chl-a in the early summer (*p* = 0.0003), but not by the late summer (*p* = 0.336). *Polynucleobacter asymbioticus* (ASV_4) was not correlated with abiotic variables in the early summer, but by the late summer, was found to be more abundant in acidic lakes (*p* = 0.010) and lakes with higher concentrations of DOC (*p* = 0.043).

Yet, because lake temperature and TDP were correlated with DOC and subsequently not included in the above analysis, we conducted follow-up analyses on the variables predicted by DOC to compare model fit between DOC, surface temperature, and TDP. The response of the key species was varied, but none were better predicted by TDP than DOC or surface temperature. Out of the alpine-associated ASVs, four were better predicted by low concentrations of DOC than low surface temperature: *Candidatus Planktophila sulfonica* (ASV_8), *Polynucleobacter asymbioticus* (ASV_4), a member of the family Oxalobacteraceae (ASV_250), and *Caulobacter* sp. (ASV_47). The abundance of five alpine-associated ASVs was better predicted by temperature than DOC: *Acinetobacter bereziniae* (ASV_288), *Flavobacterium* sp. (ASV_2), *Piscinibacter* sp. (ASV_74), *Pedobacter* sp. (ASV_82), and a member of the NS11-12 marine group (ASV_20). As for the subalpine-associated ASVs, the abundance of both *Polynucleobacter asymbioticus* (ASV_4) and *Pseudarcicella* sp. (ASV_1) was better predicted by higher concentrations of DOC than warmer surface temperatures.

## Discussion

**Figure 8:**
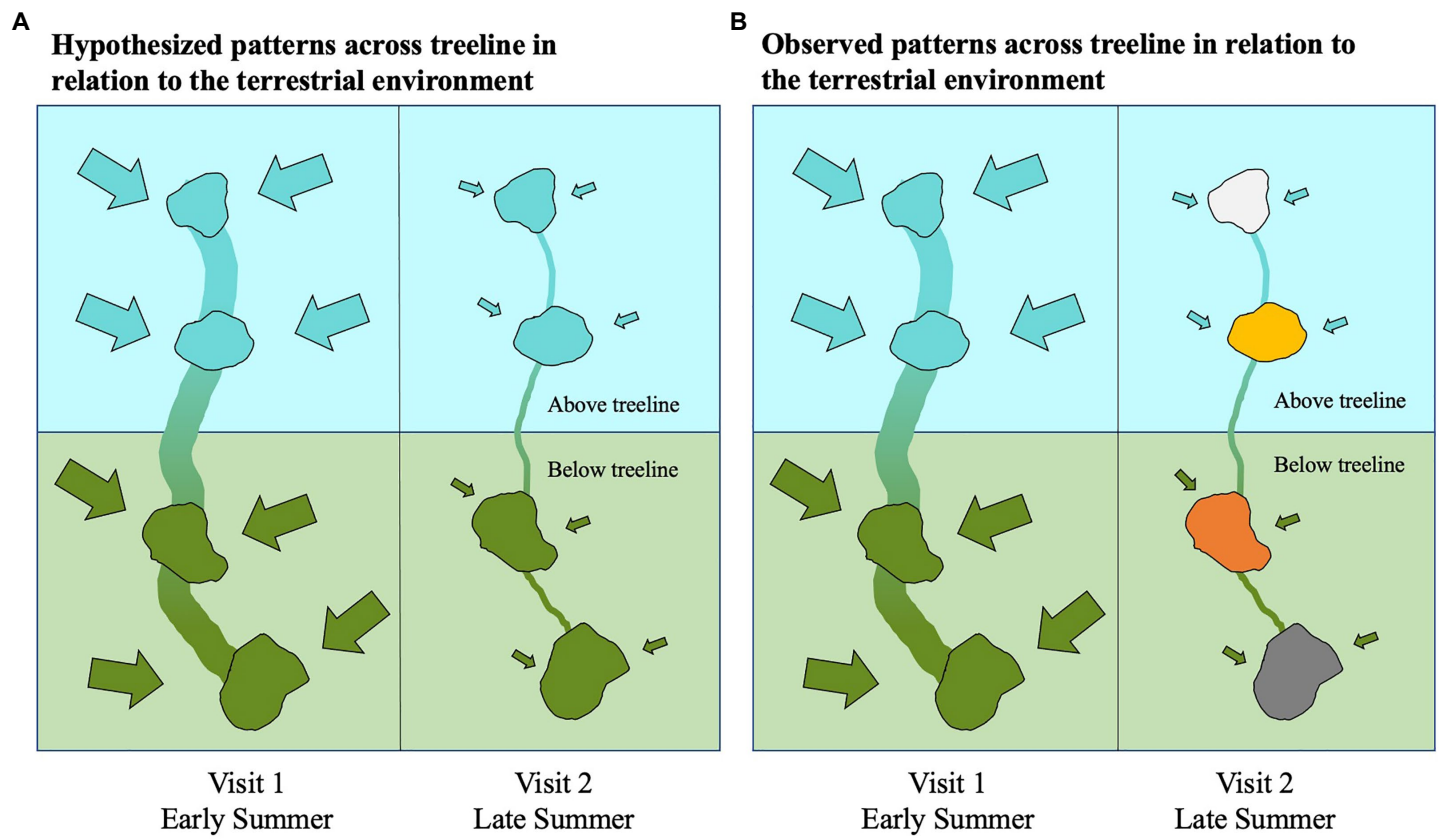
Conceptual diagram showing two potential scenarios of hydrologic influences on bacterial community composition between alpine and subalpine lakes. Our original hypothesis **(A)** displays the early summer visit on the left with high hydrologic connectivity both between lakes and with the terrestrial environment represented by a thick vertical stream line connecting the lakes and large inflow arrows. Lower inter-lake connectivity and hydrologic connection with the terrestrial environment hypothesized for the late summer visit are depicted on the right with a thinner vertical stream line and smaller inflow arrows. Blue is indicative of alpine community conditions, and green is indicative of subalpine (below treeline) conditions. The alternate hypothesis **(B)** is based on the observed patterns found in this study. The early summer visit depicted on the left, shows bacterial communities of alpine and subalpine lakes represent the same dissimilarity as their surrounding terrestrial environments when hydrologic connectivity is high. Yet, by the late summer when hydrologic connectivity is low both between lakes and with the terrestrial environment, the community composition of the lakes no longer reflect the differences found in the terrestrial environment.

In the early summer, bacterioplankton community composition of the studied alpine and subalpine lakes are distinct and reflect the community differences of terrestrial microbes across treeline. Yet, by the end of the summer, these bacterioplankton communities are no longer distinct from one another (Figure 8). These results were contrary to our original hypothesis that communities would be distinct in both the early and late summer (Figure 8A). Our study was not designed as a seasonal study, but the differences between the early and late summer visits are indicative of seasonality playing more of a role in structuring communities in lakes across treeline than we had originally anticipated. Bacterial community composition changes over time within an individual lake have been documented (Shade et al., 2007). But this is the first study, to our knowledge, to compare bacterial community composition across treeline at multiple time points. The results presented here pave the road for future comprehensive seasonal studies that compare community composition across treeline.

The observed patterns of bacterioplankton community distinction across treeline only in the early summer may point to terrestrial-aquatic connections driving composition. While the governing processes behind watershed hydrology are complex and vary based on factors, such as topology, soil quantity and quality, and vegetation (Beven and Kirkby, 1979; Ambroise et al., 1996), the seasonality of hydrologic connectivity in mountain basins is well documented (Hauer et al., 1997; Bales et al., 2006). Spring snowmelt flushes solutes, nutrients, and terrigenous microbes into lakes (Denning et al., 1991; Creed et al., 1996; Tsai et al., 2008; Fellman et al., 2009; Wilkinson et al., 2013). As summer progresses and basins dry, connectivity within a watershed decreases dramatically (Phillips et al., 2011). In addition to connections with the terrestrial environment, between-lake connectivity is also higher during spring as snowmelt flows rapidly from one lake down to the next. It is conceivable that streamflow could connect bacterioplankton communities between lakes resulting in less community divergence across treeline within a watershed. But during the early summer visit when streamflow was highest, we observed distinct communities across treeline within the same watershed. Thus, during the early summer, the connectivity with the local terrestrial environment appears to play a larger role in shaping communities than between-lake connectivity (Figure 8).

Another potential explanation of the differences in composition across treeline found between early and late summer is the role of seasonal stratification (the layering of lakes by temperature and chemistry). Because deeper lakes in this area are likely to stratify in the late summer (Lewis, 1983) and bacterial community composition often diverges with stratification (Vila et al., 1998; Shade et al., 2008), it is likely that bacterial communities of the epilimnion (surface layer of a stratified lake) would differ from the hypolimnion (bottom layer of a stratified lake). Yet, because we mixed the surface and bottom samples for each lake, we are unable to make comparisons of bacterial community composition within the water column. A large variance can also be cause for in an insignificant finding (lack of difference between alpine and subalpine lakes), and mixing the surface and bottom samples of microbially stratified lakes would increase the variance of the community composition. However, it is unlikely that stratification or the variance caused by mixing stratified surface and bottom samples is driving the patterns observed between alpine and subalpine lakes because lake depth did not differ across treeline (*p* > 0.05), and both shallow and deep lakes were represented across alpine and subalpine treatments. While stratification may not be playing a role in shaping the patterns observed across treeline, additional evidence of the terrestrial environment playing a role can be found when examining the communities at the ASV (amplicon sequencing variant) level and their associations with the terrestrial environment.

Some of the alpine-associated ASVs in our study may have been sourced from glaciers or permanent snowfields that are found around the majority of the upper study lakes. The abundance of the alpine-associated *Flavobacterium* sp. (ASV_2) from the present study shared 100% identity with a bacterium identified from a glacier in northwestern China (unpublished, NCBI-BLAST accession no. MH174130.1). Two other alpine-associated bacteria, *Sediminibacterium* sp. (ASV_7) and *Caulobacter* sp. (ASV_47), shared 100% identity with a sequence identified from the meltwater of Arctic and sub-Arctic glaciers (Kohler et al., 2020). The connection with meltwater from glaciers and permanent ice fields may be a significant driver of alpine lake bacterioplankton community in these lakes, but further research is necessary to confirm.

Overall community composition does not seem to be solely driven by abiotic factors measured in this study. Rather it appears more likely that certain bacteria are favored in specific conditions, and differences in their abundance ultimately contribute to the overall community composition (as seen in Newton and McMahon, 2011). When chemical factors alone are driving differences, concentrations will parallel temporal patterns of community composition over time. Thus, if the measured abiotic factors alone were driving the community composition differences we observed between the early and late summer, we would expect to see the same patterns for the abiotic factors as we do with community composition. However, none of the abiotic variables exhibited differences between alpine and subalpine lakes only at the early summer visit (Figure 6). For example, pH played a significant role in the community dissimilarity between alpine and subalpine lakes, but the acidity of lakes did not differ across treeline. This points to pH selectively inhibiting (or enhancing) a few bacterial species that influence the overall community composition, rather than driving community composition as a whole. One example may be the greater abundance of the alpine-associated *Caulobacter* sp. (ASV_47) in acidic lakes. Alpine lakes were not more acidic than subalpine lakes overall, but *Caulobacter* sp. (ASV_47) had a greater abundance in alpine lakes that were more acidic than others (the association for the genus *Caulobacter* with acidic environments is congruent with existing research by Percent et al., 2008). The genus *Caulobacter* has also been found to be associated with oligotrophic environments (Wilhelm, 2018) and may explain why *Caulobacter* sp. (ASV_47) was more abundant when TDN was low, and a low concentration of DOC was a better predicter of abundance than low lake temperature.

Even DOC, the abiotic factor, that was most correlated with bacterial community dissimilarity out of all of the abiotic factors, was only correlated with the abundance of two of the ASVs contributing most to community composition; the abundance of *Polynucleobacter asymbioticus* (ASV_4) was greater in lakes with higher DOC concentrations, while the abundance of *Candidatus Planktophila sulfonica* (ASV_8) was greater in lakes with lower DOC concentrations. But existing research could not confirm either of these findings. *Polynucleobacter asymbioticus* is indicated as cosmopolitan bacteria without specific habitat preferences (Hahn et al., 2009). However, the four sites sampled in Hahn et al. (2009) were much lower in elevation than our lakes and likely have DOC concentrations similar or higher than the subalpine lakes in our study. Additionally, the clade in which *Candidatus Planktophila sulfonica* belongs is notoriously difficult to culture (Kang et al., 2017), resulting in a dearth of known information and the inability to confirm or deny the preference for lakes low in DOC that we observed.

At the same time, total DOC within a lake is comprised of a pool of complex carbon molecules originating from both within the lake (autochthonous) and from the terrestrial environment (allochthonous). Allochthonous DOC has a higher concentration of aromatic carbon compounds than autochthonous DOC and has a strong seasonal aspect, with a spike during spring snowmelt (Hood et al., 2003; Miller and McKnight, 2015). Allochthonous DOC is also higher in subalpine lakes than alpine lakes (Rose et al., 2015), but we did not measure the various qualities of DOC that make up the pool of total DOC in the present study. Thus, species with an association with total DOC may be in fact reacting to the DOC quality within the lake. *Polynucleobacter asymbioticus* (ASV_4), a species we found in higher abundance in lakes with higher DOC, is known to be associated with lakes dominated by DOC composed of humic substances where it is believed that they feed on low-molecular-weight substrates remaining after the photooxidation of humic substances (Hahn et al., 2012). Species belonging to the genus *Acidovorax* have also been found to be associated with the degradation of aromatic carbon compounds (Hutalle-Schmelzer et al., 2010; Díaz et al., 2013). While the correlation with subalpine lakes was not significant at the *p* < 0.05 level for *Acidovorax* sp. (ASV_6) in the present study (*p* = 0.09), the relatively low value of *p* is worthy of noting as the lack of significant association might simply be a reflection of a low sample size. More research is warranted to verify, but the higher concentrations of allochthonous DOC in subalpine lakes (Rose et al., 2015) may be contributing to the association of these ASVs with subalpine lakes.

Not all of the ASVs correlated with the community dissimilarity were also correlated with the physiochemical factors tested (Table 3). This may be indicative of untested abiotic factors or biotic factors, such as dispersal, competition, and predation playing a role in structuring community composition. Several studies have implicated terrigenous microbe dispersal to aquatic systems as actively shaping bacterioplankton communities (Mašín et al., 2003; Lindström et al., 2006; Crump et al., 2007; Ruiz-González et al., 2015, 2017; Hayden and Beman, 2016). Knowing that the microbial communities of alpine and subalpine terrestrial environments differ (Kernaghan and Harper, 2001; Thébault et al., 2014) and the communities of our alpine lakes differed from subalpine lakes when terrestrial-aquatic connections were high, it is quite possible that terrigenous microbes are playing some sort of role in structuring the community patterns we observed. These terrigenous microbes can alter the assembly of aquatic bacterial communities through community filters, such as predation, resource competition, by acting as a source population, or becoming a food source for resident microbes.

Gendron et al. (2019) found support for strong microbial community filters in a study of upland soils, inlet and outlet streams, and lake water columns in drainage just south of RMNP. Bacterial communities of inlet streams were more similar to upland soil communities than lake water column or outlet streams were to soil communities, and lake water column communities were more similar to outlet streams than the inlet streams. Nelson (2009) also examined bacterial community composition between inlet streams and lakes in the California Sierra Nevada and found headwater inlet streams were consistently distinct from all downstream samples, suggesting that within-lake species sorting occurs in the headwater lake before downstream flow. The lack of difference between communities across treeline by the end of the summer could be a reflection of “cosmopolitanism” or the prevalence of particular bacteria ubiquitous in lakes across regions as discussed in Sommaruga and Casamayor (2009).

Yet, the lack of community distinction between alpine and subalpine lakes at the end of the summer could also be a reflection of between-lake dispersal playing a larger role than dispersal from the terrestrial environment. As long as flowing water is present in connector streams, as was the case in the present study watersheds, bacterioplankton can disperse from alpine to subalpine lakes and assemble into similar communities across treeline (Fodelianakis et al., 2014). Rain events throughout the summer are a regular occurrence in these mountains and can increase streamflow dramatically (Yang et al., 2016). Hydrologic connectivity within a watershed in the early season is controlled by precipitation and snowmelt; whereas in the late season, only precipitation is playing a role. It is possible that the spring snowmelt carries more terrestrial debris across the soil, whereas late summer precipitation washes into streams with less terrestrial debris. Yet, the top three alpine-associated ASVs remained more abundant in alpine lakes than subalpine lakes on the late summer visit and are thus not simply being washed downstream by late summer.

Because of the limited access to the study lakes, we sampled all lakes in a watershed within 1 week of ice-off of the uppermost lake in the watershed, capturing the natural differences in growing season length that alpine and subalpine lakes experience at one time. However, time since ice-off could influence bacterioplankton communities, especially snow-inhabiting or phototrophic species. For example, we found the abundance of the alpine-associated *Piscinibacter* sp. (ASV_74) to be higher in the early summer shortly after ice-off than in the late summer. Because the genus *Piscinibacter* has been found to be abundant in snow samples (Luo et al., 2020), it might have simply flowed into the lake with snowmelt and then after a certain amount of time, did not thrive in the lake environment. Thus, the abundance may have been higher in subalpine lakes as well shortly after ice-off in the late winter/early spring when the lakes were not accessible. Additionally, lakes covered with ice will generally accumulate a snowpack that inhibits the penetration of light into the water column. For example, the relative abundance of phototrophic Cyanobacteria increased in alpine lakes from the early summer to the late summer visit (Figure 3A), but did not change in subalpine lakes (Figure 3C). This may indicate that Cyanobacteria population growth had already peaked in subalpine lakes with the additional time from ice-off, whereas alpine lake Cyanobacteria growth was just beginning. Yet, overall chl-a concentrations did not differ between the early summer and the late summer visit for alpine or subalpine lakes (Figure 6C). Thus, while the timing of ice-off may have an effect on some phototrophic taxa, it is not the sole driver of community composition differences.

In conclusion, our results demonstrate that bacterioplankton community composition of the studied alpine lakes differed from subalpine lakes in the early summer, coinciding with the increased hydrologic connections of spring snowmelt. By the end of the summer, when terrestrial-aquatic connections are low, they were no longer distinct across treeline. Community composition divergence across treeline was correlated with DOC, pH, chl-a, and TDN (in order of decreasing strength). The physiochemical factors tested generally did not impact the community composition uniformly. Rather, certain bacteria were favored in specific conditions and differences in abundance of specific bacterial ASVs ultimately impacted the overall community composition. In the context of a changing climate, careful consideration of how terrestrial-aquatic connections are changing over time is imperative to preserving the unique bacterial communities of alpine lakes. And further, because lakes are like drains, changes in the terrestrial environment can impact bacterial communities potentially altering higher trophic levels and biogeochemical processing of the lake.

## Data Availability Statement

The datasets generated for this study can be found at: https://portal.edirepository.org/nis/mapbrowse?packageid=edi.900.2 and https://www.ncbi.nlm.nih.gov/Traces/study/?acc=PRJNA604620.

## Author Contributions

KV designed the study and secured the funding and wrote the original draft manuscript. KV and AS collected and processed the samples in the laboratory of SS. KV, EG, and HH-M contributed to data analysis and interpretation. All authors revised the original draft manuscript for intellectual content and contributed to the article and approved the submitted version.

## Funding

This work was funded through a Rocky Mountain National Park cooperative agreement (#P14AC00749). Publication of this article was funded by the University of Colorado Boulder Libraries Open Access Fund.

## Conflict of Interest

The authors declare that the research was conducted in the absence of any commercial or financial relationships that could be construed as a potential conflict of interest.

## Publisher’s Note

All claims expressed in this article are solely those of the authors and do not necessarily represent those of their affiliated organizations, or those of the publisher, the editors and the reviewers. Any product that may be evaluated in this article, or claim that may be made by its manufacturer, is not guaranteed or endorsed by the publisher.

## Footnotes

^1^ https://github.com/leffj/mctoolsr/
